# A secondary data analysis of Internet use in caregivers of persons with dementia

**DOI:** 10.1002/nop2.2

**Published:** 2014-07-25

**Authors:** Heejung Kim, Karen M. Rose, Richard G. Netemeyer, Elizabeth I. Merwin, Ishan C. Williams

**Affiliations:** ^1^ University of KansasSchool of Nursing3901 Rainbow Boulevard Mail Stop 4043Kansas CityKansas66160; ^2^ University of VirginiaSchool of Nursing202 Jeanette Lancaster WayCharlottesvilleVirginia22908‐0782; ^3^ Ralph A. Beeton Professor of Free EnterpriseUniversity of Virginia McIntire School of CommerceRobertson HallRoom 509 P.O. Box 400173CharlottesvilleVirginia22904; ^4^ Duke University School of Nursing3077A Pearson Building307 Trent Dr.DUMC 3322DurhamNorth Carolina27710

**Keywords:** Caregivers, dementia, internet, resources, stress

## Abstract

**Aim:**

This paper is a secondary data analysis to investigate relationships among caregiver stress appraisal, self‐rated health and health‐related Internet use.

**Design:**

Cross‐sectional correlation design.

**Methods:**

National Alliance for Caregiving telephone survey conducted in the USA was a primary data source collected in 2009 from 258 caregivers of persons with dementia, who used the Internet to perform care‐giving tasks. Based on Pearlin's Stress Process Model, structural equation modelling was conducted.

**Results:**

Caregivers with poor health reported higher levels of caregiver stress appraised, which was associated with more Internet use for health‐related purposes. It is required to develop effective Internet‐based resources to meet the needs of highly stressed caregivers of persons with dementia. However, there was no relationship between self‐rated health and health‐related Internet use in dementia caregiver.

## Introduction

The rapid growth of the Internet and other technology in recent years has significantly increased the distribution of health information and resources to a much wider healthcare consumer than was previously possible. Almost 80% of Internet users and 59% of US adults are seeking health/medical information on the Internet (Fox 2012). Among them, informal caregivers reported a greater use of online health information, support groups and emailing compared with non‐caregivers (Chou *et al*. 2011, Fox & Brenner 2012). In addition, caregivers are more likely to participate in social networks, especially when they are seeking health information, care or support (Fox & Brenner 2012). This higher rate can be explained by their intense interest in and positive perceptions of Internet‐based approaches and positive outcomes for better health and caregiving (Eysenbach 2003, Kinnane & Milne 2010, Lewis *et al*. 2010, Fox & Brenner 2012).

Use of Internet‐based resources for health‐related purposes (hereafter referred to as health‐related Internet use) is an important activity to informal caregivers of persons living with dementia (hereafter referred to as dementia caregivers) while performing care‐giving tasks. Dementia caregivers are known as one of the most vulnerable caregiver populations. They are experiencing higher levels of stress, care‐giving intensity and diverse health problems covering a range of physical, psychological, social and spiritual aspects (Zarit 2006, Schulz & Sherwood 2008, Family Caregiver Alliance 2009). Previous intervention studies based on an Internet‐based care modality reported positive outcomes including better health status (Marziali & Garcia 2011) and increasing levels of confidence in decision‐making, positive caregiver gain, self‐efficacy and intention to seek help, as well as a more positive attitude towards caregiving (Beauchamp *et al*. 2005, Powell *et al*. 2008, Lewis *et al*. 2010).

### Background

Dementia caregivers have reported several important benefits of the Internet‐based modality of care, which they found to be convenient and useful because of its high accessibility regardless of time and place (Beauchamp *et al*. 2005). Using traditional formal services and resources in the community is difficult for them because of time‐constrained situations due to constant care, requiring another caregiver while they are absent, or due to financial burdens (Toseland *et al*. 1999, Beeber *et al*. 2008, Powell *et al*. 2011). Thus, they reported benefits while using Internet‐based information and support resources (Lewis *et al*. 2010).

Health‐related Internet use in these dyads dealing with dementia leans towards indirect use made by caregivers. Indirect internet use is defined as providing their care recipient with online information and resources when actions are requested by or on behalf of the ill care recipient, while direct use is performed for the user's own benefit (Eysenbach 2003, Kinnane & Milne 2010). Indirect use by caregivers most often occurs when their care recipients lack the necessary knowledge or skills to do so, are far less likely to be cured of their disease and may be too ill to directly use the Internet (Kinnane & Milne 2010). Cognitive impairment and functional disability (Qiu *et al*. 2009) inevitably hinders direct health‐related Internet use by individuals with dementia.

However, health‐related Internet use in non‐experimental settings has rarely been assessed as a resource‐use behaviour in dementia caregivers in contrast to other types of caregivers, particularly those caring for family members with cancer (Eysenbach 2003, Kinnane & Milne 2010). Previous studies have used interventional study designs because they considered the Internet as an alternative modality for delivering healthcare (Beauchamp *et al*. 2005, Lewis *et al*. 2010). Most studies of resource use have focused on the efficacy of traditional formal resources such as health and human resources (Toseland *et al*. 1999, 2002) or community‐based services (Beeber *et al*. 2008) rather than web‐based resources. Thus, it is unclear how caregivers' initiatives regarding health‐related Internet use are associated with both health and caregiving in daily living. To address the gaps identified above, this study aimed to provide an in‐depth description of this topic, extending beyond mere descriptions of Internet user profiles (National Telecommunications & Information Administration 2011, Powell *et al*. 2011). Our research question was ‘Are there significant relationships among caregiver stress appraisal, health‐related Internet use and self‐rated health?'

This study was guided by the Stress Process model (Pearlin *et al*. 1990), which was specifically designed to understand dementia caregiving. This framework emphasizes the multidimensional nature of care‐giving stress consisting of five constructs: (1) care‐giving context; (2) primary stressors; (3) secondary stressors; (4) resources; and (5) outcomes. Care‐giving context includes the socio‐demographic status of the care recipients and their caregivers, as well as the care‐giving history. Stressors include symptoms or impairments of the care recipients and subjective experiences of caregivers that have a negative impact on health or well‐being of dyads of persons with dementia and their caregivers. Resources are mediators that explain how caregivers may present their burden differently even when dealing with the same stressors; caregivers will experience more stress if they perceive the demands being placed on them as being beyond their coping resources. Outcomes include physical, emotional, psychological and spiritual issues in caregivers. This study proposes that health‐related Internet use was a behaviour to use care‐giving resources based on the Stress Process Model (Pearlin *et al*. 1990).

## The Study

### Design

This is a cross‐sectional and correlational study using a secondary data analysis. In this study, the Stress Process Model (Pearlin *et al*. 1990) directed the design of the hypothesized model, guided variable selection of caregiver stress in measured data and assisted data interpretation in the final model.

### Method

#### Description of primary data source

The primary data provided by the National Alliance for Caregiving and the American Association of Retired Persons (NAC/AARP) database were collected from March–June in 2009 using a standardized computerized‐telephone interviewing system. Interviews were conducted with 6806 adults living in the states of California, Delaware, Illinois, Kansas, Ohio, Virginia and the state of Washington. Random sampling was conducted in general, but African American, Asian and Hispanic groups, as well as older adults (age 50 years or older) were oversampled. The survey collected demographic, health‐related and care‐giving information about both the caregivers and their care recipients. The NAC/AARP database was selected for the study because: (1) it provides up‐to‐date information on Internet use by dementia caregivers; (2) it has used methodologically verified sampling and data collection since 1997 (NAC/AARP 2009a,b).

#### Sample

A total of 258 dementia caregivers who used the Internet during caregiving were selected. Dementia caregivers were defined as persons ‘providing unpaid care or assistance to a family member, relative, friend, or anyone who had Alzheimer's dementia, other types of dementia, or dementia‐related conditions.’ Unpaid care was defined as ‘helping with personal needs or household chores without any financial compensation (NAC/AARP 2009b, p. 3).’ Exclusion criteria were: (1) the care recipients reported as being younger than 18 years of age as dementia is a rare disease in children (Qiu *et al*. 2009); (2) caregivers who did not report any information about the dementia condition of the care recipient; and (3) caregivers who never used the Internet seeking health and care‐giving resources.

#### Measures

Caregiver stress was measured based on subjective appraisal regarding physical strain, emotional stress and financial hardship. They were measured based on a 5‐point Likert scale. Higher scores indicated worse physical strain, emotional stress and financial hardship for caregivers. Across the three items, moderate correlations were observed ranging from 0·42–0·53 (Table 1) with acceptable reliability (Cronbach's α = 0·70).

**Table 1:** Correlation coefficients for the measured variables.

Variables	1	2	3	4	5	6
1. Physical strain	1·00					
2. Emotional stress	0·52**	1·00				
3. Financial hardship	0·53**	0·42**	1·00			
4. Self‐rated health	−0·29**	−0·27**	−0·29**	1·00		
5. Diverse types of Internet use	0·18**	0·24**	0·30**	−0·09	1·00	
6. Frequency of Internet use	0·19**	0·24**	0·28**	−0·13*	0·55**	1·00

*P < 0·05. **P < 0·01.

Self‐rated health is an indicator used to assess the health status of caregivers. Respondents answered a single question, ‘In general, would you say your health is: 1 = poor; 2 = fair; 3 = good; 4 = very good; and 5 = excellent’ extracted from the Medical Outcomes Study 36‐Item Short‐Form General Health Survey (Ware & Sherbourne 1992). This simple measure has been found to be a valid and reliable measure and is commonly used as an index of health and well‐being for dementia caregivers in both interventional design (Mittelman *et al*. 2007, Elliott *et al*. 2010) and health and human service‐use studies (Toseland *et al*. 1999, 2002).

Health‐related Internet use can be defined as using the Internet with the purpose of improving health, well‐being and caregiving (Eysenbach 2003, Kinnane & Milne 2010). Both frequency and diverse types of Internet use were used to construct health‐related Internet use. Frequency was scored on a 4‐point Likert scale based on a single question: ‘How often, if at all, have you gone to Internet websites in the past year to find information in any way related to being a caregiver for your care recipient? Often, sometimes, rarely, or never? (NAC/AARP 2009b, p. 18).’ Higher scores indicated more frequent Internet use as reported by caregivers. Diversity of use was a summed score of six items related to the care recipient's condition, services, support group, a specific task, healthcare providers and care facilities (range: 0–6). Higher scores indicated more diverse use of health‐related Internet information during caregiving (Pearson *r *=* *0·55, *P *<* *0·01, Cronbach's α = 0·82).

#### Statistical analysis

**Figure 1:**
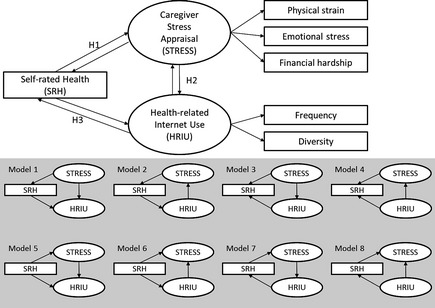
Hypothesized models and eight competing models.

A structural equation modelling (sem) tested the hypothesized relationships among: a latent variable of caregiver stress appraisal, a latent variable of health‐related Internet use and a measured variable of self‐rated health (Figure 1). This sem analytical technique not only allows simultaneous testing of multiple hypotheses representing complex relationships between latent variables but also accommodates multiple indicators with measurement errors (Loehlin 2004). The analysis was initiated using two separate confirmatory factor analyses (CFAs) for assessing measurement models of both latent variables of caregiver stress appraisal and health‐related Internet use. sems compared eight completing models using Mplus version 7. Model fit between hypothesized models and the measured data was examined based on chi‐square statistics, comparative fit Index (CFI), Tucker‐Lewis index (TLI), root mean square error of approximation, (RMSEA) and its associated 90% confidence interval (90% CI), Akaike information criterion (AIC) and Bayesian information criterion (BIC) (Hu & Bentler 1999, Schermelleh‐Engel *et al*. 2003). The significance‐level criterion for all statistical tests was set at α = 0·05, two‐tailed. The testing hypotheses were the following:

H1: There is a significant relationship between caregiver stress appraisal and self‐rated health.

H2: There is a significant relationship between caregiver stress appraisal and health‐related Internet use.

H3: There is a significant relationship between self‐rated health and health‐related Internet use.

Two cases were dropped from the sem based on list‐wise deletion across all measured variables. This represented only 0·8% of the sample, thus no data imputation was conducted (Meyers *et al*. 2006). All statistical assumptions were checked including univariate/multivariate normality and linearity. No transformation was required to collect univariate normality, no outliers were found concerning multivariate normality and linearity. The final sample size in sem (*N *=* *256) was deemed sufficient to conduct both CFAs and sems because there were 18 free parameters estimated in the sem and minimum required sample size was 200 (Loehlin 2004).

#### Ethical considerations

This study was a secondary analysis using de‐identified data without Health Insurance Portability and Accountability Act (HIPAA) identifiers. Exempt approval from the Institutional Review Board was obtained from the investigators' university and data use was approved by NAC/AARP.

## Results

### Sample characteristics in caregivers and their care recipients with dementia

Socio‐demographic and caregiving‐related information for the 258 caregivers and their care recipients is shown in Table 2. Persons with dementia were older adults (mean = 80·14, sd 13·20). The majority were women (68·6%) and living in home settings (52·7%). Approximately one‐fifth had Alzheimer's disease and lived with their caregivers. Dementia caregivers in our study were middle‐aged (mean = 51·95, sd 12·48), spent 28·51 hours per week on care‐giving tasks (sd 47·26) and had performed their care‐giving role for 4·89 years (sd 7·15) on average. The majority were female (64·0%), at least college‐level educated (60·5%) and not the primary caregiver (55%). Three quarters had household incomes greater than $30,000 yearly. Compared with other demographics (Toseland *et al*. 2002, National Family Caregivers Association & Family Caregiver Alliance 2006), our participants were more likely to be the dementia patients’ children (75·9%) rather than their spouses (3·9%); the sample in Toseland *et al*. (2002) consisted of 49·3% offspring and 37·0% spouses.

**Table 2:** Socio‐demographic characteristics of caregivers and their care recipients with dementia (*N *=* *258).

Description of persons with dementia	
Age, Mean (SD)	80·14 (13·20) year
Gender, *N* (%)	
Female	177 (68·6%)
Types of dementia	
Alzheimer's disease	40 (15·5%)
Non‐Alzheimer's disease	218 (84·5%)
Resident status, *N* (%)	
Home setting	136 (52·7%)
Living with the caregiver	56 (21·7%)
Description of their caregivers	
Age (year), Mean (SD)	51·95 (12·48) year
Time spent for caregiving per week, Mean (SD)	28·51 (47·26) hours
Duration of caregiving, Mean (SD)	4·89 (7·15) years
Gender, *N* (%)	
Female	165 (64%)
Primary caregiver, *N* (%)	
Yes	116 (45%)
Relationship with persons with dementia as, *N* (%)	
Spouse	10 (3·9%)
Parent	8 (3·1%)
Child or grandchild	196 (75·9%)
Relative	24 (9·3%)
Friend/ non‐relative/ neighbour	19 (7·4%)
Missing	1 (0·4%)
Education, *N* (%)	
High school or less	102 (39·5%)
Some college or higher	156 (60·5%)
Household income, *N* (%)	
Less than $30,000/year	38 (14·7%)
$30,000/year or more	193 (74·8%)
Missing data	27 (10·5%)
Subjective responses of care‐giving stress, Mean (SD)	
Physical strain	2·41 (1·27)
Emotional stress	3·20 (1·21)
Financial hardship	2·96 (0·76)
Health status, Mean (SD)	3·62 (1·05)
Diverse types of Internet use, Mean (SD)	2·73 (1·61)
Frequency of Internet use, *N* (%)	
Often	70 (27·1%)
Sometimes	108 (41·9%)
Rarely	80 (31·0%)

### Measurement model testing

All correlation coefficients of measured variables were summarized in Table 1. The CFA provided a good fit to the observed data for the latent constructs of caregiver stress appraisal and health‐related Internet use (Loehlin 2004). For model specification, regression weights were constrained with 1 on two paths: (1) from caregiver stress appraisal to physical strain; and (2) from health‐related Internet use to frequency of use. Measurement model analyses showed that caregiver stress appraisal and health‐related Internet use had one dimension with the following fit indices. Model fit indices of the confirmatory factor analysis of caregiver stress appraisal included: χ^2^ (3, *N *=* *256) = 107·46, *P *<* *0·01; CFI = 1·00; TLI = 1·00; RMSEA < 0·01. All factor loadings of the observed variables were significant (*P *<* *0·001), suggesting that the latent variables were well represented by their respective observed indicators. In addition, model fit indices of the confirmatory factor analysis of health‐related Internet use included: χ^2^ (1, *N *=* *256) = 21·53, *P *<* *0·01; CFI = 0·95; TLI = 1·05; RMSEA < 0·01. Two factor loadings of the observed variables were significant, suggesting that the latent variables were well represented by their respective observed indicators.

### Structural model testing

sems were tested to compare 8 different models due to three possible bi‐directional relationships among caregiver stress appraisal, self‐rated health and health‐related Internet use (Figure 1). To select the best fit model, all model indices were compared and summarized in Table 3. Chi‐square statistics and most of model fit indices were identical among 8 models. However, Model 5 was chosen as the final models based on AIC and BIC results. Model 5's lowest AIC and BIC indices meant superior model fit compared with those of Models 1‐4 (Hu & Bentler 1999, Schermelleh‐Engel *et al*. 2003).

**Table 3:** Summary of model fit indices.

	χ^2^(7, *N *=* *256), *P*	CFI	TLI	RMSEA	90% CI for RMSEA	AIC	BIC
Good model fit is determined if the fit indices are:	Not significant	≥0·95	≥0·95	≤0·06		Smaller compared with others	Smaller compared with others
Models 1–4 and 6–8	10·46 _(d.f. = 7),_ 0·16	0·989	0·977	0·044	0·000, 0·095	4550·30	4621·20
Model 5 (Final model)	10·46 _(d.f. = 7),_ 0·16	0·989	0·977	0·044	0·000, 0·095	3800·318	3864·13

CFI, comparative fit Index; TLI, Tucker‐Lewis index; RMSEA, root mean square error of approximation; CI, Confidence interval; AIC, Akaike information criterion; BIC, Bayesian information criterion.

**Figure 2:**
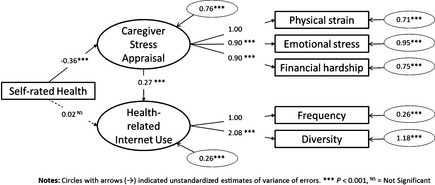
Final full structural equation model (sem). Circles with arrows (→) indicate unstandardized estimates of variance of errors. ***P < 0·001; NS, not significant.

The final model (model 5 in Figure 1) results indicated excellent model fit between hypothesized model and tested data, χ^2^ (7, *N *=* *256) = 10·46, *P *=* *0·16, CFI = 0·989, TLI = 0·989, RMSEA = 0·044 and 90% confidence interval around RMSEA < 0·001, 0·095 (Table 3). All conclusions were drawn from this model. Figure 2 displays interrelationships among caregiver stress appraisal, self‐rated health and health‐related Internet use. Higher caregiver stress appraisal was associated with poor self‐rated health (*β* = −0·36, *P *<* *0·001) and more use of Internet for health and care‐giving purposes (*β* = 0·27, *P *<* *0·001). However, there was no significant relationship between self‐rated health and health‐related Internet use (*P *=* *0·70).

## Discussion

This study identified the relationships among caregiver stress appraisal, self‐rated health and health‐related Internet use based on the Stress Process Model (Pearlin *et al*. 1990). Higher levels of caregiver stress appraised were directly associated with a poor rating of health status, which is consistent with previous findings from dementia caregiver research (Pearlin *et al*. 1990, Elliott *et al*. 2010). Although dementia caregivers with higher levels of stress were more likely to engage in health‐related Internet use, this behaviour had no apparent relationship with their health status.

Stressed caregivers’ strong health‐related Internet use can be understood in terms of resource use as a coping strategy. Caregiver stressors are facilitators to use resource use as coping strategies. Dementia caregivers with higher levels of stress are more likely to use health and human services (Toseland *et al*. 2002), which facilitate their coping mechanisms in response to stressful situations during caregiving (Eysenbach 2003, Kinnane & Milne 2010). Stress occurs when there is an imbalance where the demands of individuals or the family exceed the existing capacity to cope (Lazarus & Folkman 1984). To resolve the recognized level of stress, individuals actively seek additional coping strategies to help them resolve the mismatch between demand and capability. Thus, the increased use of the Internet for health‐related purposes in stressed caregivers reflects their strong motivation to explore all possible coping strategies.

However, our study showed an insignificant relationship between health‐related Internet use on self‐rated health. First, dementia caregivers may consider health‐related Internet use as a new care‐giving task (Kinnane & Milne 2010) rather than a health promotion behaviour that supports their own health and well‐being. Second, self‐rated health may not be sufficiently sensitive to reflect the specific demands of caregivers related to Internet use. Thus, more diverse indicators in terms of health, well‐being and caregiving should be examined to understand the comprehensive effects of health‐related Internet use, such as improved self‐efficacy, confidence as a caregiver, or empowerment (Eysenbach 2003, Beauchamp *et al*. 2005). Third, Internet‐based strategies for caregivers may not always meet their needs, which vary considerably and change according to caring responsibilities over time (Kinnane & Milne 2010).

There are several clinical implications. First, healthcare providers should consider how best to integrate Internet‐based resources into their current practice (Eysenbach 2003, Powell *et al*. 2011). Previous researchers have suggested that health‐related Internet use should be seen as a supplement to existing health service provision rather than as a total replacement for it (Powell *et al*. 2011). Caregivers still preferred healthcare providers as their primary source of care and information and considered Internet‐based information and resources as secondary or adjunct sources (Eysenbach 2003, Kinnane & Milne 2010, Powell *et al*. 2011, Fox & Brenner 2012). Practitioners should therefore look for ways to maximize the benefits of Internet‐based resources in families dealing with dementia beyond simply listing useful Internet‐based resources. Assessing psychological stress should precede the design of tailored interventions across the disease and care‐giving trajectory.

Second, nurses should be aware of the unavoidable disadvantages and challenges inherent in many Internet‐based resources and strategies. Despite the advantages and benefits the Internet offers, its users often suffer from technical difficulties, inaccurate or low‐quality information, possible misuse or confusion and the overwhelming volume of information available (Eysenbach 2003, Lewis *et al*. 2010, Powell *et al*. 2011). Nurses are often responsible for guiding dementia caregivers to qualified resources, educating them in how to use them effectively and regularly monitoring how Internet‐based strategies are impacting their care‐giving trajectory (Kinnane & Milne 2010). Quality assurance is also important to ensure consumer satisfaction and positive outcomes and thus encourage greater use of online resources (Toseland *et al*. 2002).

### Study Limitations and Future Directions

As our findings are based on self‐reported, cross‐sectional survey data with convenience sampling, this study inevitably suffers from limited inference of causality and generalizability as well as possible responder bias. Thus, future studies are highly recommended to adopt a longitudinal design using more comprehensive measurements of caregiver stress and Internet use. More detailed information of Internet use and health indicators will provide a better in‐depth understanding of how each type of Internet use influences specific outcomes in caregivers.

## Conclusion

- This study provides a better understanding of health‐related Internet use in dementia caregivers in the United States. Our findings will contribute to efforts to develop more effective Internet‐based interventions and outcome measures.
- Our results highlight that the higher the levels of self‐appraised caregiver stress facilitate greater health‐related Internet use. However, greater Internet use does not appear to be related to dementia caregiver's health status.
- Internet‐based resources could be a secondary adjunct to be used alongside conventional healthcare services in current practice regarding both actual and virtual healthcare use.
- Health care providers, including nurses specifically, are responsible for providing guidance or recommendations for specific, high‐quality, Internet‐based resources that meet caregivers’ health and psychological needs.

## Conflict of interest

No conflict of interest has been declared by the authors.

## Author contributions

All authors have agreed on the final version and meet at least one of the following criteria [recommended by the ICMJE (http://www.icmje.org/ethical_1author.html)]:

- substantial contributions to conception and design, acquisition of data, or analysis and interpretation of data;
- drafting the article or revising it critically for important intellectual content.
